# Recent Advances in the Synthesis and Applications of 1,2,3/1,2,5-Thiadiazole- and Benzo[*c*][1,2,5/1,2,3]thiadiazole-Type Compounds

**DOI:** 10.3390/molecules30224373

**Published:** 2025-11-12

**Authors:** Diego Quiroga, Daniel Rodríguez, Ericsson Coy-Barrera

**Affiliations:** 1 Bioorganic Chemistry Laboratory, Faculty of Basic and Applied Science, Universidad Militar Nueva Granada, Cajicá 250247, Colombia; ericsson.coy@unimilitar.edu.co; 2 Biological Control Laboratory, Faculty of Basic and Applied Science, Universidad Militar Nueva Granada, Cajicá 250247, Colombia; daniel.rodriguez@unimilitar.edu.co

**Keywords:** benzothiadiazole derivatives, fluorescent probes, bioimaging agents, thiadiazole derivatives, Hurd–Mori reaction

## Abstract

Thiadiazole derivatives, such as 1,2,3-thiadiazole, 1,2,5-thiadiazole, benzo[*c*][1,2,5]thiadiazole, and benzo[*d*][1,2,3]thiadiazole, have garnered significant attention due to their exceptional chemical and physical properties. These molecules, which contain sulfur and nitrogen atoms in their heterocyclic structure, have a variety of applications in agriculture, materials, and pharmaceuticals. In this review, we examine the most commonly used synthetic methods for these compounds, with a focus on the most recent techniques, including green synthesis, solid-phase chemistry, and catalytic processes, which enable greater efficiency, improved selectivity, and reduced environmental impact. Advances in the structural modification of these molecules to improve their photophysical properties and biocompatibility are also discussed. Finally, we highlight future research directions and emerging applications of thiadiazole derivatives across molecular medicine, nanotechnology, and agriculture, underscoring their potential to revolutionize multiple scientific and technological fields.

## 1. Introduction

Thiadiazoles and benzothiadiazoles are a group of heterocyclic compounds that have been widely explored for their fascinating properties and applications across various fields [1]. These heterocyclic compounds contain sulfur and nitrogen atoms in their structure. Although benzothiadiazoles share the same basic skeleton of a thiadiazole ring, they differ in the relative position of the sulfur and nitrogen atoms within the heterocyclic ring, which influences their chemical and physical properties [2]. The most representative heterocyclic system of this family corresponds to 1,3,4-thiadiazole **1**, which has gained enormous relevance in modern pharmacological research due to its remarkable antidiabetic activity [3], as has been widely demonstrated in various studies. In recent years, multiple investigations have highlighted their ability to act on different key enzymes in carbohydrate metabolism, such as α-glucosidase, α-amylase, protein tyrosine phosphatase 1B (PTP1B), and sodium-dependent glucose cotransporters (SGLT2) [4,5,6]. For instance, hybrid carbazole-1,3,4-thiadiazole derivatives have been shown to exhibit potent inhibition against α-amylase and α-glucosidase, with IC_50_ values of 0.68 µM and 0.14 µM, respectively, significantly outperforming the standard drug acarbose [7]. Similarly, indazole-1,3,4-thiadiazoles showed outstanding inhibition of α-glucosidase and thymidine phosphorylase, with favorable ADMET profiles [8]. Likewise, 1,3,4-thiadiazole Schiff-type derivatives were developed as SGLT2 inhibitors, demonstrating inhibitory activity comparable to dapagliflozin and a significant glucose-lowering effect in vivo [9]. Other studies have reported indole-1,2,3-triazole-1,3,4-thiadiazole hybrids with outstanding α-glucosidase inhibitory activity (IC_50_ = 31.91 μM), which is markedly lower than that of acarbose (844.81 μM), highlighting their strong therapeutic potential [10]. Similarly, the structural combination of indole and 1,3,4-thiadiazole yielded derivatives with potent α-amylase inhibitory activity, again surpassing acarbose [11]. Further advances include 5*H*-[1,2,4]triazino[5,6-b]indole-1,3,4-thiadiazole derivatives showing IC_50_ values as low as 2.5 µM against α-glucosidase, approximately fifteen times more active than acarbose [12]. Additionally, 1,3,4-thiadiazolyl-1,3-thiazolidine-2,4-dione compounds have demonstrated potent PTP1B inhibition and proven in vivo efficacy in lowering blood glucose levels [13]. At the mechanistic level, these compounds promote insulin sensitivity by interacting with the PPAR-γ receptor, as observed with amide and urea derivatives of 1,3,4-thiadiazole, whose effects were comparable to those of the drug pioglitazone [14]. Finally, the ability of nitrothiadiazolopyrimidines to inhibit the formation of advanced glycation end products (AGEs) and the enzyme DPP-4 demonstrates that 1,3,4-thiadiazoles also contribute to preventing vascular complications associated with diabetes [15].

**Figure 1:**
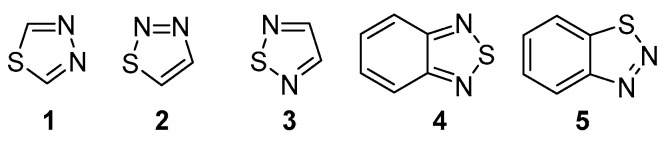
Generic structures of (benzo)thiadiazole derivatives such as 1,3,4-thiadiazole **1**, 1,2,3-thiadiazole **2**, 1,2,5-thiadiazole **3**, benzo[*c*][1,2,5]thiadiazole **4**, and benzo[*d*][1,2,3]thiadiazole **5**.

The above background consolidates 1,3,4-thiadiazoles **1** as a versatile and promising structural scaffold for the design of new antidiabetic therapies, with multiple mechanisms of action, high inhibitory potency, and encouraging safety profiles. However, 1,2,3-thiadiazole **2**, 1,2,5-thiadiazole **3**, benzo[*c*][1,2,5]thiadiazole **4**, and benzo[*d*][1,2,3]thiadiazole **5** represent also highly interesting compound classes with similar heterocyclic moiety (Figure 1), specifically due to the achieved development associated with their chemical synthesis. **2**-Type heterocycle features a continuous N = N-S arrangement, while **3** has an alternate structure in terms of the distribution of the heteroatomic atoms (Figure 1). On the other hand, **4** and **5** correspond to fused variants with different ring arrangements, leading to distinct stability and reactivity characteristics [16,17].

Building on previous research and the potential of heterocyclic compounds across various applications, this review summarizes recent developments in the synthesis and use of compounds containing 2–5 heterocyclic moieties over the last decade. The review describes key progress in this area, with a focus on their roles in organic chemistry, molecular electronics, and biomedicine. It also considers current challenges and possible future directions, which may help guide further research and application of these compounds in emerging technologies and therapeutic strategies across related scientific fields.

## 2. Synthesis and Medicinal and Pharmaceutical Chemistry Applications

Heterocycles **2**–**5** are widely used in bioimaging, fluorescence, and diagnostics due to their exceptional optical properties, such as the ability to emit light in the visible spectrum, making them ideal for applications in cellular research and molecular medicine [18]. Furthermore, their structure enables the creation of highly sensitive sensors and biosensors, particularly in biochemistry and pharmacology, where they are used to detect contaminants and biomarkers [19,20,21,22,23]. In agriculture, these molecules are explored as bioactive compounds to improve disease resistance and protect crops. Nanotechnology also benefits from these structures, given their potential to interact at the molecular level, improving drug delivery systems and the development of advanced materials. Thus, differences in the chemical structures of these molecules determine their versatility and applications across a wide range of scientific disciplines. For instance, **4**-type compounds have emerged as an innovative class of fluorescent probes with up-and-coming biological applications [19]. In particular, the identification of novel **4**-based probes for the selective detection of lysosomal pH and mercury (Hg^2+^) has further expanded their applicability in monitoring pathological processes, including lipid metabolism dysfunctions and the endoplasmic reticulum stress response [20,21]. The development of fluorescent organic nanoparticles and metal–organic frameworks based on benzothiadiazoles has emerged as a promising tool in bioimaging, drug delivery, and the design of sensitive fluorescent sensors [22,23].

**Scheme 1:**
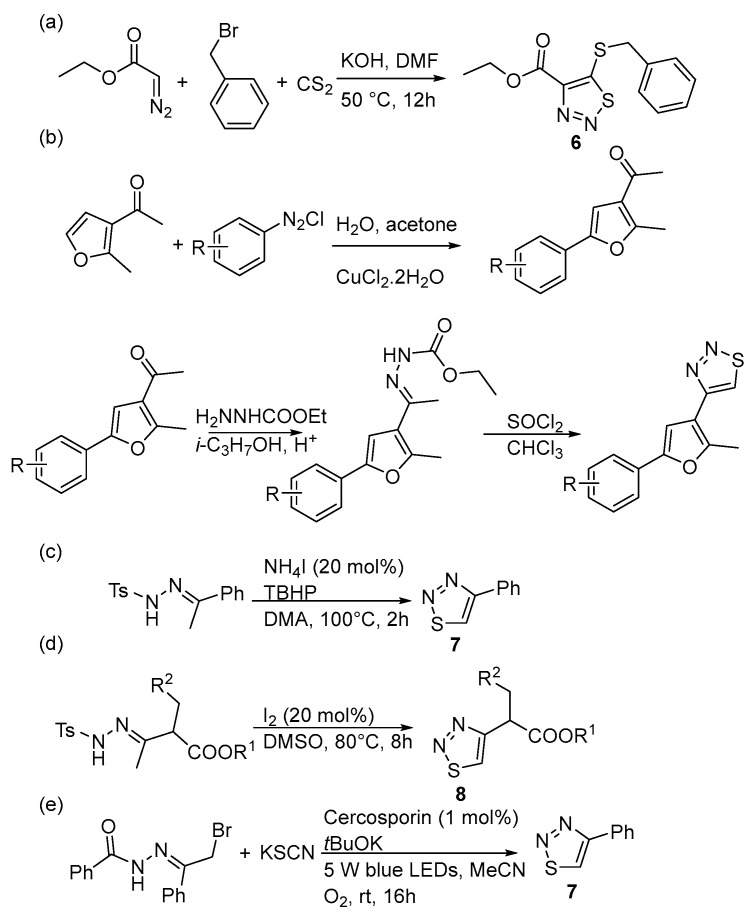
Representative synthetic methods for 1,2,3-thiadiazole derivatives. (**a**) Nucleophilic addition of α-diazocarbonyl compounds to carbon disulfide; (**b**) Arylation of 2-methyl-3-acetylfuran via the Gomberg–Bachmann reaction followed by Hurd–Mori cyclization; (**c**) Ammonium iodide-catalyzed cycloaddition of *N*-tosylhydrazones with elemental sulfur in the presence of TBHP; (**d**) Iodine-catalyzed reaction of ketone-derived *N*-tosylhydrazones with elemental sulfur; (**e**) Photocatalytic synthesis of thiadiazoles from benzohydrazides and KSCN using perylenequinonoid pigments as green catalysts under mild conditions.

Given the potential applications of these compounds, the development of novel synthetic methods remains a widely explored area. The synthesis of heterocyclic compounds containing the thiadiazole nucleus is carried out through various strategies, many of which rely on traditional ring-forming reactions, nucleophilic substitution, and organometallic techniques [24,25,26]. One of the most prominent approaches in the synthesis of 1,2,3-thiadiazoles is the nucleophilic addition of α-diazocarbonyl compounds to carbon disulfide (Scheme 1a).

This method, carried out under mild conditions and using inexpensive reagents such as carbon disulfide, has proven to be highly efficient and versatile. Precursors such as ethyl diazoacetate (EDA) and benzyl bromide (BnBr) participate in the reaction, which is carried out in a dimethylformamide (DMF) medium at 50 °C for 12 h. The yield of the desired product, ethyl 5-(benzylthio)-1,2,3-thiadiazole-4-carboxylate **6**, reaches 81%. This approach has been noted for its operational simplicity and for its ability to apply reaction conditions to a wide variety of compounds [27].

Another notable method is the arylation reaction of 2-methyl-3-acetylfuran, achieved via the Gomberg-Bachmann reaction, which results in the formation of 1,2,3-thiadiazole derivatives (Scheme 1b). This process is based on the arylation of substituted furans and the subsequent transformation of carboethoxyhydrazones into thiadiazoles via the Hurd–Mori reaction. The incorporation of electron-withdrawing groups on the aryl ring increases the thermal stability of the obtained products, suggesting their potential for use in pharmaceutical materials and products [28]. A recent strategy employed ammonium iodide (NH_4_I) as an inexpensive catalyst to facilitate the cycloaddition of *N*-tosylhydrazones with elemental sulfur, in the presence of *tert*-butyl hydroperoxide (TBHP) as an organic oxidant (Scheme 1c). This method enables the production of 4-aryl-1,2,3-thiadiazoles **7** from a wide range of *N*-tosylhydrazones, demonstrating excellent tolerance to various functional groups and facilitating gram-scale synthesis. The main advantage of this approach lies in the avoidance of inorganic oxidants, making it a more sustainable and environmentally friendly process [29]. The iodine-catalyzed reaction of ketone-derived *N*-tosylhydrazones has been carried out under mild conditions with elemental sulfur (Scheme 1d). This process produces 4-alkyl- and 4-styryl-1,2,3-thiadiazoles **8**, which have potential applications in pharmacology and agriculture. This method is characterized by its efficiency, high yields, selectivity, and excellent tolerance to a wide range of functional groups [30]. Recently, photocatalysis with perylenequinonoid pigments, such as cercosporine, has been explored for the synthesis of thiadiazoles **7** from benzohydrazides and KSCN (Scheme 1d). This process is environmentally friendly, conducted under mild conditions, and exhibits excellent compatibility with various functional groups, enabling the synthesis of thiadiazole derivatives with potential applications in drug development [31].

**Scheme 2:**
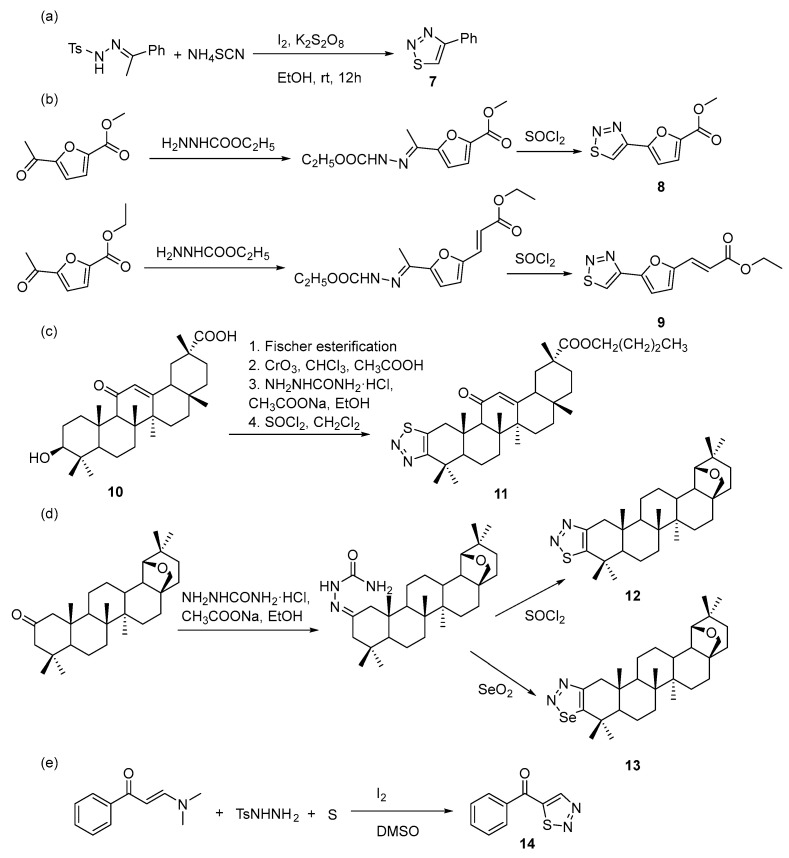
Selected synthetic strategies for 1,2,3-thiadiazole derivatives. (**a**) Room-temperature synthesis from *N*-tosylhydrazones and NH_4_SCN in ethanol; (**b**) Hurd–Mori cyclization of furan-derived hydrazones; (**c**) Synthesis of a glycyrrhetinic acid–based 1,2,3-thiadiazole via semicarbazone intermediates; (**d**) Preparation of 1,2,3-thiadiazole and 1,2,3-selenadiazole triterpenoid derivatives through Hurd–Mori and Lalezari reactions; (**e**) Metal-free cyclization of enaminones, tosylhydrazine, and sulfur.

An efficient method for synthesizing 1,2,3-thiadiazoles uses *N*-tosylhydrazones and ammonium thiocyanate (NH_4_SCN) as precursors, with ethanol serving as an environmentally friendly solvent at room temperature. This process exhibits broad substrate scope and demonstrates good tolerance to functional groups, with product yields ranging from 53% to 87% depending on the reaction conditions and substrates employed (Scheme 2a). The compound 4-phenyl-1,2,3-thiadiazole **7** has been successfully used in Sonogashira and Suzuki–Miyaura acylations, yielding 62% and 74%, respectively [32]. In another approach, ethyl (*E*)-2-(1-(5-(methoxycarbonyl)furan-2-yl)ethylidene)hydrazine-1-carboxylate and ethyl (*E*)-2-(1-(5-((*E*)-3-ethoxy-3-oxoprop-1-en-1-yl)furan-2-yl)ethylidene)hydrazine-1-carboxylate (previously obtained from methyl and ethyl 5-acetylfuran-2-carboxylate, respectively), undergo the Hurd–Mori reaction to form methyl 5-(1,2,3-thiadiazol-4-yl)furan-2-carboxylate **8** and ethyl (*E*)-3-(5-(1,2,3-thiadiazol-4-yl)furan-2-yl)acrylate **9** (Scheme 2b). This process allowed yields around 69%, with the added advantage that the thiadiazole fragment remains stable even under thermal conditions [33]. The synthesis of a 1,2,3-thiadiazole derivative of glycyrrhetinic acid **10** was performed via a characterized semicarbazone intermediate. The synthetic route involves esterification of **10**, oxidation to a 3-oxo derivative, formation of a semicarbazone, and subsequent Hurd–Mori cyclization (Scheme 2c). This method offers an 85% yield in the formation of compounds beneficial for pharmacological research, particularly in the development of antitumor agents [34]. The synthesis of 1,2,3-thiadiazole **12** and 1,2,3-selenadiazole **13** derivatives from triterpenoids such as allobetulone and 2-oxoallobetulin has also been reported. The condensation reaction, followed by the Hurd–Mori reaction and the Lalezari method, yields compounds with high regioselectivity (Scheme 2d). These compounds exhibit antimicrobial and anti-inflammatory properties, making them of great interest in medicinal chemistry [35]. The use of triterpenoids, such as dipterocarpol and hollongdione, in Hurd–Mori reactions enables the production of 1,2,3-thiadiazole derivatives fused to these compounds. The application of these derivatives in the development of new drugs is promising, particularly given their biological properties, including anti-inflammatory, antibacterial, and antifungal effects [36]. An additional approach involves the synthesis of 5-acyl-1,2,3-thiadiazoles **14** via a cyclization reaction between enaminones, tosylhydrazine, and elemental sulfur (Scheme 2e). This transition metal-free process provides products in good yields and is compatible with a wide range of functional groups [37].

### 2.1. Antibacterial, Antiviral, and Antifungal Activity

**Scheme 3:**
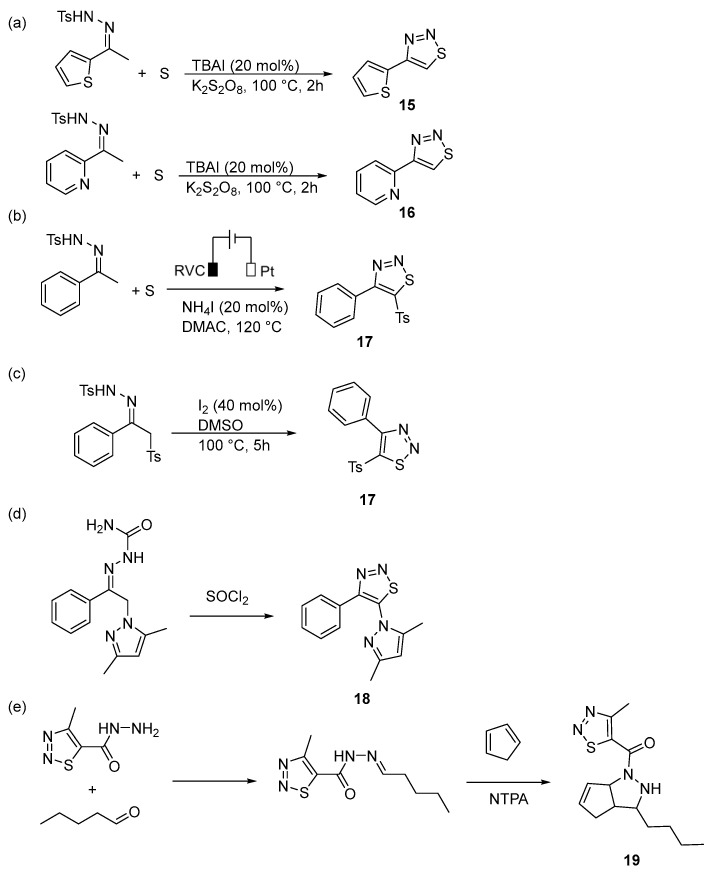
Recent synthetic methods for 1,2,3-thiadiazole derivatives. (**a**) TBAI-catalyzed, metal-free synthesis from *N*-tosylhydrazones and sulfur under mild conditions; (**b**) Electrochemical, metal- and oxidant-free synthesis of 1,2,3-thiadiazoles from sulfur and *N*-tosylhydrazones; (**c**) Formation of 4-phenyl-5-tosyl-1,2,3-thiadiazoles from 2-(*p*-toluenesulfonyl)-*N*-tosylhydrazones and S_8_; (**d**) Synthesis of bioactive pyrazolyl-1,2,3-thiadiazoles via Hurd–Mori cyclization of pyrazolyl semicarbazones; (**e**) [3 + 2] Cycloaddition between hydrazones and cyclopentadiene catalyzed by *N*-trifluorophosphoramide (NTPA), yielding cyclopentapyrazole–thiadiazole hybrids.

1,2,3-Thiadiazoles and their derivatives remain of great interest in both basic and applied research, with a range of biological activities and applications in medicine, agriculture, and materials science. Recent advances in their synthesis methods, along with improvements in efficiency and sustainability, open up new prospects for their development and commercialization in various fields. 1,2,3-Thiadiazole derivatives, which can be synthesized from *N*-tosylhydrazones and elemental sulfur [37,38,39], are known for their applications in both the pharmaceutical and materials manufacturing fields and exhibit diverse biological activities, including antibacterial, antiviral, antitumor, antifungal, and herbicidal properties as plant growth regulators. These compounds are of particular interest due to their versatility in various industrial and biomedical sectors. A tetrabutylammonium iodide (TBAI)-catalyzed reaction between *N*-tosylhydrazones and sulfur has been developed, enabling the synthesis of 1,2,3-thiadiazole compounds in moderate to good yields. This metal-free method represents an accessible and efficient alternative for producing thiadiazoles, improving the process known as the Hurd–Mori reaction under mild conditions in the presence of TBAI as a catalyst (Scheme 3a) [38]. The products obtained include compounds such as 4-(thiophen-2-yl)-1,2,3-thiadiazole **15** and 4-(pyridin-2-yl)-1,2,3-thiadiazole **16**, which are of particular interest in medicinal chemistry due to their pharmacological properties and potential for the development of new drugs.

Mo et al. reported the importance of a new metal- and oxidant-free electrochemical method for the synthesis of 1,2,3-thiadiazoles **7** from elemental sulfur and *N*-tosylhydrazones, characterized as a more sustainable alternative method that also reduces the risks associated with traditional methods, which require highly reactive reagents and excessive oxidants (Scheme 3b) [39]. The electrochemical reaction was carried out in a three-necked flask using a cross-linked glassy carbon anode and a platinum cathode, with *N*-tosylhydrazones, sulfur, and NH_4_I as catalysts in *N*,*N*-dimethylacetamide (DMAC) at 120 °C. The constant current applied was 10 mA for approximately 6 h. Optimization of the reaction conditions enabled yields of up to 85% in the formation of 1,2,3-thiadiazoles **7**. Evaluation of the compatibility of different substituents on the *N*-tosylhydrazones revealed that electron-donating groups favored higher yields. This development represents a significant advance in green synthetic chemistry, offering an efficient and safe route to produce these compounds. 1,2,3-Thiadiazoles could be obtained using simple, commercially available substrates and I_2_ and CuCl_2_ as promoters [40]. The compound 4-phenyl-1,2,3-thiadiazole **7** was obtained in 75% yield, demonstrating the versatility of the method and allowing its application to a wide range of substrates, with yields ranging from 71% to 89%. Compounds such as 4-phenyl-5-tosyl-1,2,3-thiadiazole **17**, have been reported as an intermediate of great relevance in organic chemistry [40]. Recently, an efficient method for the synthesis of **17** from 2-(*p*-toluenesulfonyl)-*N*-tosylhydrazones with S8 was reported (Scheme 3c). The results showed yields of 24–65% for the desired products, with a significant improvement in selectivity upon adjusting the reaction conditions. This study demonstrated that the reaction is compatible with a wide range of functional groups [41].

Pyrazolyl-1,2,3-thiadiazole derivatives **18** are compounds with a broad spectrum of biological activities, including antibacterial, antiviral, anticancer, fungicidal, herbicidal, and neuroprotective properties. The synthesis of **18** was carried out by a reaction between pyrazolyl phenylethanones and semicarbazide hydrochloride in the presence of sodium acetate and methanol, to form semicarbazones, which were then subjected to cycloaddition with thionyl chloride under Hurd–Mori conditions (Scheme 3d) [42]. Several pyrazolyl-1,2,3-thiadiazole derivatives were obtained with yields ranging from good to excellent [42]. The development of novel cyclopentapyrazoles containing a 1,2,3-thiadiazole ring, with applications as antifungal agents via [3 + 2]-cycloaddition reaction between hydrazones and cyclopentadiene, using an *N*-trifluorophosphoramide (NTPA) catalyst, allowed the preparation of compound **19** (Scheme 3e). Optimization of the reaction conditions, including time, temperature, and solvent, resulted in a remarkable yield of the products, characterized by outstanding antifungal activity, notably when compounds **19** and (4-methyl-1,2,3-thiadiazol-5-yl)(3-propyl-3,3a,4,6a-tetrahydrocyclopenta[*c*]pyrazol-1(2*H*)-yl)methanone were combined, which showed 100% inhibition against *Botrytis cinerea*. EC_50_ values of 6.37 μg/mL and 13.85 μg/mL were obtained for *B. cinerea* and *Fusarium culmorum*, respectively. These results underscore the potential of cyclopentapyrazoles as effective fungicidal agents, warranting further study in their development [43].

**Scheme 4:**
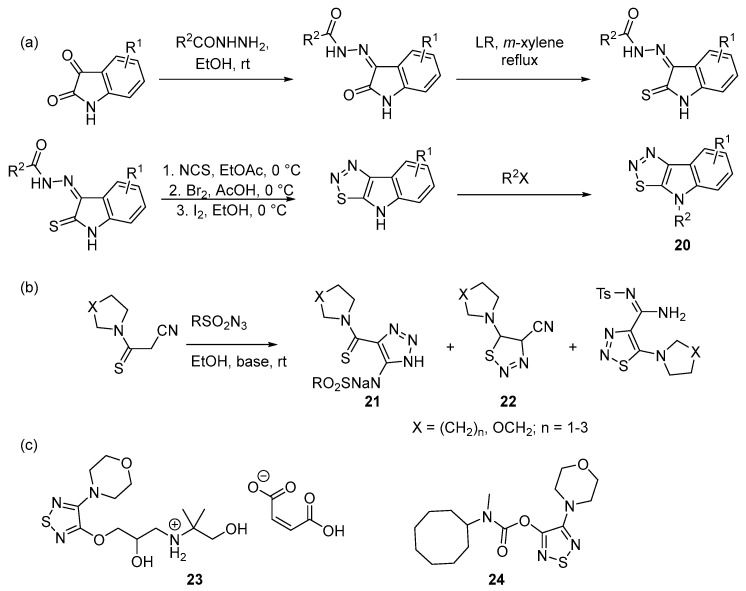
Synthetic approaches to fused and functionalized 1,2,3-thiadiazole derivatives. (**a**) Oxidative cyclization of 3-hydrazonoindoline-2-thiones with halogenating agents (NCS, Br_2_, I_2_) to yield 4-substituted-4*H*-[1,2,3]thiadiazolo[5,4-*b*]indoles; (**b**) Base-dependent cyclization of 2-cyanothioacetamides with sulfonyl azides leading selectively to 1,2,3-thiadiazoles or 1,2,3-triazoles; (**c**) Chemical structures of biologically active compounds **23** and **24**.

Regarding 4-substituted-4*H*-[1,2,3]thiadiazolo [5,4-*b*]indole derivatives **20**, Belskaya et al. reported an efficient synthetic route to these compounds (Scheme 4a), which exhibit potential antiproliferative activity, suggesting their potential application in cancer treatment. The proposed oxidative cyclization method constitutes a more efficient and versatile alternative than the classical Wolff–Barley method for the synthesis of *N*-substituted 4*H*-1,2,3-thiadiazolo [5,4-*b*]indoles **20**. Starting from 3-hydrazonoindoline-2-thiones, oxidation with halogenated agents (NCS, bromine, or iodine) afforded 4*H*-[1,2,3]thiadiazolo [5,4-*b*]indoles in high yields (70–91%) as single, well-defined products. Furthermore, [1,2,3]thiadiazolo[5,4-*b*]indol-2-ium intermediate salts were isolated, confirming the proposed mechanism of thiadiazole ring formation via intramolecular attack and subsequent elimination of the ester group. Subsequently, it was demonstrated that 4*H*-[1,2,3]thiadiazolo[5,4-*b*]indoles can be selectively functionalized at the nitrogen atom of the indole ring, by alkylation, acylation, and sulfonylation reactions, to generate a series of *N*-substituted 4*H*-1,2,3-thiadiazolo[5,4-*b*]indoles **20** in good yields. These results confirm the efficacy, selectivity, and applicability of the new synthetic approach for the preparation of potentially bioactive thiadiazoloindole heterocycles. A key feature of this approach is its smooth execution, yielding the target compounds in excellent isolated yields (70–91%), offering a significant advantage over previously reported methods [44].

Compounds obtained from the reaction of azides with 2-cyanothiacetamides have demonstrated a wide range of applications in medicinal chemistry, such as bioconjugates, chemosensors, and materials chemistry. These compounds (which include derivatives of 1,2,3-triazoles **21** obtained via intramolecular cyclization, and thiadiazoles **22** obtained via intermolecular cyclization) were synthesized with yields ranging from 57% to 93%. The results obtained demonstrated that the synthesis of 1,2,3-triazoles or 1,2,3-thiadiazoles from 2-cyanothioacetamides and sulfonyl azides depends strongly on the reaction conditions, in particular the type and amount of base used, as well as the nature of the solvent. It was observed that the use of nitrogenous bases (pyridine, triethylamine, DBU) in aprotic media or in ethanol results in the exclusive formation of 1,2,3-thiadiazoles in good yields (69–90%). In contrast, the use of a strong base, such as sodium ethoxide, in an ethanolic medium allows directing the reaction towards 1,2,3-triazoles. A high amount of sodium ethoxide and low temperatures (−10 °C) favor the production of 1,2,3-triazoles in high yields (up to 83%), avoiding significant formation of (*E*)-5-substituted-*N*’-tosyl-1,2,3-thiadiazole-4-carboximidamide side products. Kinetic and NMR follow-up studies confirmed that 1,2,3-triazoles are generated from intermediate 1,2,3-thiadiazoles, suggesting a sequential conversion under strongly basic conditions. This method is presented as an efficient and economical alternative to producing these compounds, expanding the available routes in organic chemistry and potentially favoring their use in various technological and biomedical applications (Scheme 4b) [45].

Compounds containing the 1,2,5-thiadiazole ring exhibit a wide variety of biological activities because this heterocycle acts as a hydrogen bonding domain and a two-electron donor system, in addition to constituting a structurally restricted pharmacophore. In this context, the compound 2-((2-hydroxy-3-((4-morpholino-1,2,5-thiadiazol-3-yl)oxy)propyl)amino)-2-methylpropan-1-ol maleate **23** demonstrated the biological potential of this class of compounds. This compound exhibited a crystalline nature and showed in vitro antibacterial activity, along with a slight capacity for DNA cleavage. Molecular docking, ADMET, and molecular dynamics studies revealed strong interactions of **23** with the target 3IVX (docking score of −8508 kcal/mol), superior to those of the control, ciprofloxacin (−3879 kcal/mol). Furthermore, in silico analysis predicted that the compound is non-carcinogenic and non-toxic according to the Ames test, with an estimated human oral absorption of 69.639%. In microbiological assays, it exhibited moderate inhibition against *Mycobacterium tuberculosis* (MIC = 25.00 µg/mL) and moderate activity against *Staphylococcus aureus*, *Bacillus* spp., *Klebsiella pneumoniae*, and *Escherichia coli*. These results support the potential of the 1,2,5-thiadiazole ring for the development of new antimicrobial agents with improved pharmacological profiles [46]. Furthermore, carbamate derivatives of 1,2,5-thiadiazoles have demonstrated significant activity as inhibitors of the enzyme α/β-hydrolase domain 6 (ABHD6), a relevant therapeutic target in inflammatory processes and metabolic disorders. 4-Morpholino-1,2,5-thiadiazol-3-yl cyclooctyl(methyl)carbamate **24** was identified as a potent and irreversible inhibitor of hABHD6, with an IC_50_ value of 44 nM and an approximate 230-fold selectivity over FAAH and LAL enzymes. Furthermore, activity-based protein profiling assays indicated that **24** exhibits high selectivity for serine hydrolases in the murine brain membrane proteome. These properties confirm that 1,2,5-thiadiazole derivatives can serve as a basis for the design of novel, selective ABHD6 inhibitors with potential applications in the treatment of obesity and type II diabetes [47].

### 2.2. Antitumoral and Anticancer Activity

**Scheme 5:**
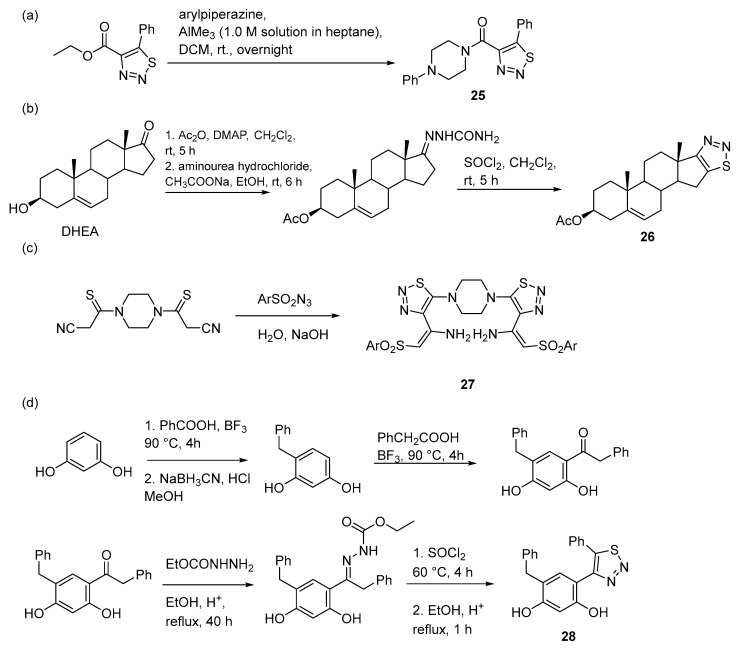
Synthetic routes and representative structures of 1,2,3-thiadiazole derivatives with potential anticancer activity. (**a**) Design and synthesis of XRP44X analogs **25** as microtubule-destabilizing agents; (**b**) preparation of DHEA-based thiadiazole derivatives **26** with potential antitumor properties; (**c**) synthesis of 1,2,3-thiadiazol-4-carbimidamides **27** via thioamide–azide coupling; (**d**) synthesis of 4-benzyl-6-(5-phenyl-1,2,3-thiadiazol-4-yl)benzene-1,3-diol **28** as a selective Hsp90 inhibitor.

5-Aryl-4-(4-arylpiperazine-1-carbonyl)-1,2,3-thiadiazole **25** derivatives have demonstrated potential applications as microtubule-destabilizing agents in anticancer activity (Scheme 5a).

Wang et al. focused their experiments on the design and synthesis of new XRP44X analogs, which are considered microtubule-destabilizing agents relevant to cancer therapy. Structural modifications were performed starting from the structural model of **25** (Scheme 5a), and their antiproliferative activities were evaluated using MTT assays in various cancer cell lines, including SGC-7901, A549, and HeLa. In addition, tubulin polymerization experiments and immunofluorescence analysis were conducted to investigate the effects on microtubule dynamics. The main results indicate that some compounds exhibited potent antiproliferative activities, with an IC_50_ value of 0.092 ± 0.005 μM against the HeLa cell line. This compound also inhibited tubulin polymerization and altered microtubule dynamics [48].

Dehydroepiandrosterone (DHEA), which is being investigated for its potential antitumor activity, was used as a precursor for derivatives fused with thiadiazoles **26**, which could have applications in cancer therapy. Acetylation reactions and thiadiazole formation using DHEA and other reagents under controlled conditions, such as a nitrogen atmosphere and specific temperatures (Scheme 5b). The biological activity of some of these compounds was evaluated, revealing notable antitumor potential [49]. Filimonov et al. reported reactions of thioamides with azides in aqueous media to yield 1,2,3-thiadiazol-4-carbimidamides **27** and 1,2,3-triazole-4-carbothioamides through a one-step, atom-economical, and environmentally friendly process [50]. Finally, 4-benzyl-6-(5-phenyl-1,2,3-thiadiazol-4-yl)benzene-1,3-diol **28** was obtained and is being investigated as an isoform-specific inhibitor of Hsp90, with significant potential for cancer treatment—a novel approach to the synthesis of selective Hsp90 inhibitors was discussed. The synthesis was carried out in several steps, starting with the Friedel–Crafts acylation of 1,3-benzenediol, followed by the reduction of the carbonyl groups, formation of hydrazones, and finally Hurd–Mori cyclization to obtain the thiadiazoles (Scheme 5d). Compounds were synthesized in moderate to good yields and that they exhibited promising potential to be evaluated as Hsp90 inhibitors, which could have significant implications in cancer therapy [51].

**Scheme 6:**
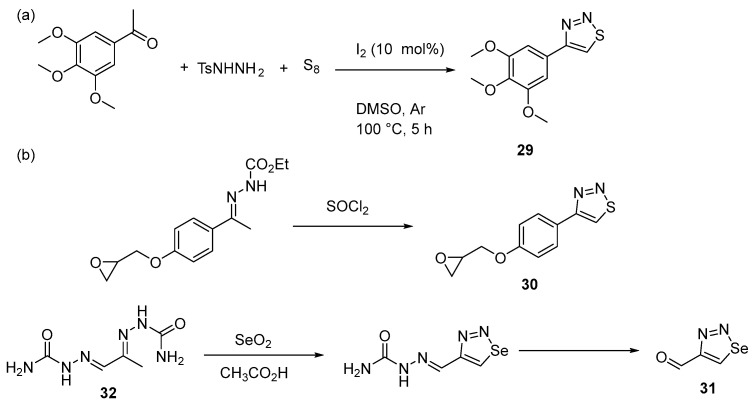
Synthetic approaches to 1,2,3-thiadiazole and 1,2,3-selenadiazole derivatives. (**a**) Iodine-catalyzed reaction of *N*-tosylhydrazones with elemental sulfur in DMSO; (**b**) Synthesis of 1,2,3-thiadiazole **30** via treatment of hydrazone intermediates with thionyl chloride, and preparation of 1,2,3-selenadiazole **31** through condensation of methylglyoxal with semicarbazide.

Li et al. reported the reaction of *N*-tosylhydrazones with sulfur to afford 1,2,3-thiadiazoles. *N*-tosylhydrazones can be readily prepared from *p*-toluenesulfonhydrazide and ketones, making this an accessible route to the synthesis of organic compounds. The reaction between *N*-tosylhydrazones and sulfur was carried out under optimized conditions using I_2_ as a catalyst in DMSO (Scheme 6a). The main results show yields ranging from 70% to 97% for the formation of the desired products, demonstrating the method’s high efficiency and applicability at the gram scale. The synthesized compound **29** acted as a neuroprotective reagent and was obtained in 85% yield [52].

Three 1,2,3-thiadiazole and 1,2,3-selenadiazole derivatives **30** and **31** were synthesized via the conversion of the corresponding ketone to hydrazone intermediates. The 1,2,3-thiadiazole derivative **30** was produced by treating hydrazone with thionyl chloride, and 1,2,3-selenadiazole derivative **31** was synthesized starting from methylglyoxal, which was transformed into methylglyoxal *bis*(semicarbazone) **32** through condensation with semicarbazide. The selenadiazole **31** was then obtained by reacting **32** with selenium dioxide in glacial acetic acid (Scheme 6b). These compounds were evaluated for their antimicrobial and antitumor activities, demonstrating significant action against various pathogens. Compounds **30** (a propenoxide derivative) and **31** (a carbaldehyde derivative) showed activity against the fungus *Candida albicans*, while **30** also showed effectiveness against the Gram-negative bacterium *Escherichia coli*. Regarding antitumor activity, compound **31** showed activity against several tumor cell lines, including SW480, HCT116, C32, MV3, HMT3522, and MCF-7. The results suggest that 1,2,3-thiadiazole and 1,2,3-selenadiazole derivatives have significant potential as antimicrobial and antitumor agents. However, further investigation is required to understand the cellular mechanisms underlying their cytotoxic activity. This breakthrough is particularly relevant given the growing problem of antibiotic resistance and the need for new therapeutic agents in the fight against cancer [53].

### 2.3. New Therapeutic and Pharmaceutical Agents

**Scheme 7:**
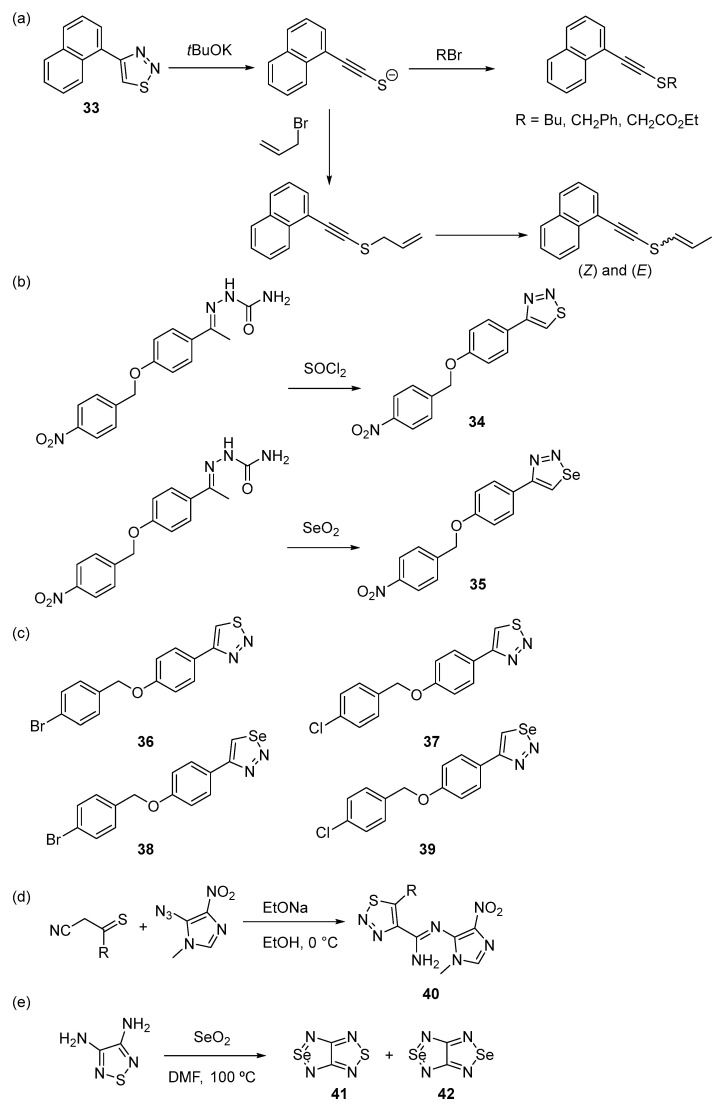
Synthetic methodologies involving 1,2,3- and 1,2,5-thiadiazole/selenadiazole derivatives. (**a**) Use of 4-(1-naphthyl)-1,2,3-thiadiazole **33** as precursor of 1-naphthylacetylene sulfides; (**b**) Preparation of compounds **34**–**35**; (**c**) Chemical structures of compounds **36**–**39**; (**d**) Synthesis of amidines **40** containing thiadiazole–triazole frameworks by reaction of thiocetamide with azides; (**e**) Conversion of 1,2,5-thiadiazoles into 1,2,5-selenadiazoles **41**–**42** via reaction with SeO_2_.

4-Phenyl-1,2,3-thiadiazole-type compounds have been known for their significant applications in the pharmaceutical industry and as intermediates in the synthesis of various organic compounds. A novel method for the synthesis of 1-naphthylacetylene sulfides by the decomposition of 4-(1-naphthyl)-1,2,3-thiadiazole **33** with potassium *tert*-butoxide in THF was reported by Yekhlef (Scheme 7a), demonstrating its use as an affordable precursor [54]. This compound was obtained from 1-naphthylmethylketone by treating its ethoxycarbonylhydrazone with thionyl chloride. When 4-(1-naphthyl)-1,2,3-thiadiazole **33** was treated with potassium *tert*-butoxide in anhydrous THF, it readily decomposed with the evolution of nitrogen to yield potassium 2-(1-naphthyl)ethynethiolate. Subsequent alkylation of the reaction mixture with an excess of alkyl halide produced butyl, benzyl, and ethylacetyl 2-(1-naphthyl)-1-ethynyl sulfides. Alkylation of potassium 2-(1-naphthyl)ethynethiolate with an excess of allyl bromide afforded a mixture of *Z*- and *E*-isomers of 2-(1-naphthyl)-1-ethynyl-1-propenylsulfide.

Compounds such as **34** and **35** demonstrated broad-spectrum activity, including the ability to combat *Pseudomonas aeruginosa* infections. This research highlights the usefulness of 1,2,3-thiadiazole and 1,2,3-selenadiazole derivatives as potential antibacterial agents, suggesting the need for further studies to optimize their efficacy in clinical applications [55]. New 1,2,3-thiadiazole and 1,2,3-selenadiazole derivatives, including 4-[4-((4-bromobenzyl)oxy)-phenyl]-1,2,3-thiadiazole **36**, 4-[4-((4-chlorobenzyl)oxy)-phenyl]-1,2,3-thiadiazole **37**, 4-[4-((4-bromobenzyl)oxy)-phenyl]-1,2,3-selenadiazole **38**, and 4-[4-((4-chlorobenzyl)oxy)-phenyl]-1,2,3-selenadiazole **39** (Scheme 7c), were synthesized and evaluated in vitro for their antimicrobial activity against various pathogenic bacterial strains, showing notable efficacy against both Gram-positive and Gram-negative bacteria, with particular emphasis on compounds **37** and **38**. Furthermore, compounds **36** and **38** were evaluated for in vivo genotoxicity in rats by measuring 8-hydroxy-2′-deoxyguanosine (8-OHdG), a marker of DNA damage. The results showed that compound **38** significantly reduced urinary 8-hydroxy-2′-deoxyguanosine levels by 50.2%, indicating low genotoxicity, whereas **36** increased such levels. In terms of toxicity, the median lethal dose (LD_50_) values for both compounds were greater than 5000 mg/kg body weight, indicating a suitable safety profile. These findings suggest that 1,2,3-thiadiazole and 1,2,3-selenadiazole derivatives, with their potent antimicrobial activity and low toxicity, could have great potential as antibiotic agents, opening the door to their future therapeutic application in the treatment of bacterial infections [56].

Belyaev reported an innovative and efficient method for synthesizing amidines **40**, which combine thiadiazole and triazole structures and could contribute to the development of new bioactive compounds. The synthesis was carried out by dissolving sodium in absolute ethanol, then adding thiocetamide and azide. The resulting mixture was kept at 0 °C for a specified time, then filtered and the final product purified (Scheme 7d). The results indicated that the reaction between the precursors yielded amidines with yields of 58–76%. The described method represents an atom-efficient strategy for the synthesis of novel heterocyclic compounds [57]. 1,2,5-Selenadiazoles could be obtained from 1,2,5-thiadiazoles by reaction with selenium dioxide (Scheme 7e). Konstantinova et al. reported that 1,2,5-thiadiazoles fused with electron-withdrawing heterocycles, such as 1,2,5-thiadiazole, 1,2,5-selenadiazole, quinoxaline, and thiadiazolopyrazine, did not react with SeO_2_ in common organic solvents such as chloroform, benzene, THF, ethanol, or 1,4-dioxane, under reflux. However, in dimethylformamide (DMF), the reaction was initiated at 80–100 °C, and after prolonged heating, the corresponding *mono*- and *bis*-1,2,5-selenadiazoles (**41**–**42**) were obtained in good yields [58].

## 3. Applications in Agriculture

**Scheme 8:**
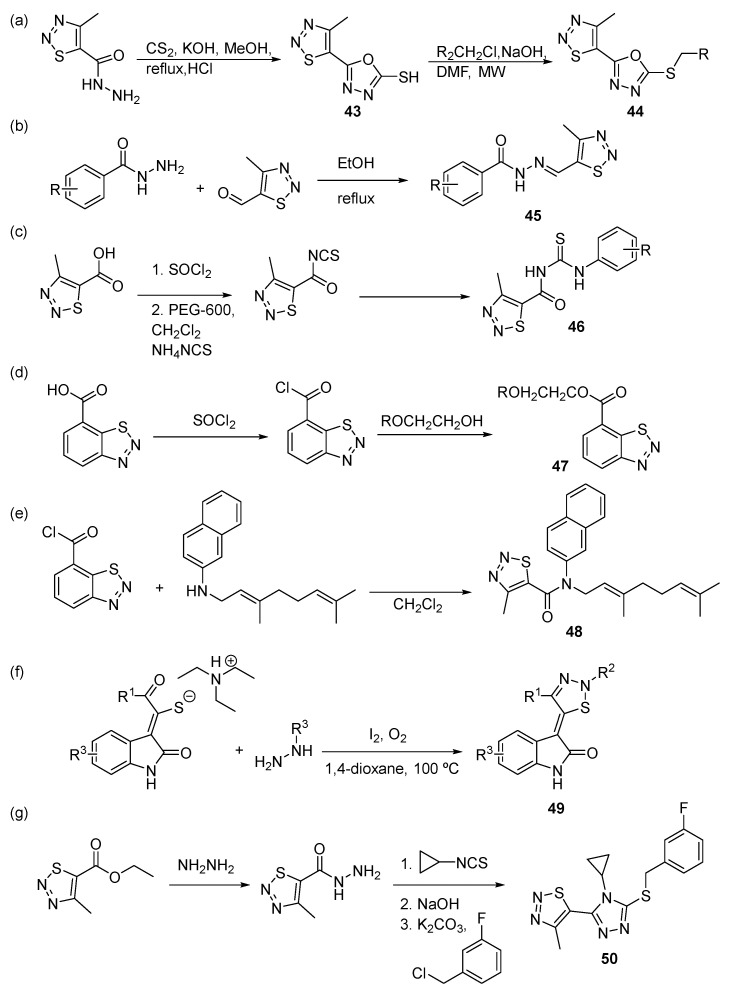
Synthetic strategies for multifunctional 1,2,3-thiadiazole-based derivatives. (**a**) Preparation of 5-(4-methyl-1,2,3-thiadiazol-5-yl)-1,3,4-oxadiazole-2-thiol **43** and related derivatives **44**; (**b**) Synthesis of 4-methyl-1,2,3-thiadiazole-5-carboxaldehyde benzoyl hydrazones **45** through condensation of corresponding aldehydes with substituted benzoyl hydrazides; (**c**) Solid–liquid phase-transfer catalyzed synthesis of *N*-(substituted phenyl)-*N*′-(4-methyl-1,2,3-thiadiazol-5-yl)thioureas **46** using PEG-600 as a catalyst; (**d**) Synthesis of benzo-1,2,3-thiadiazole-7-carboxylic acid derivatives **47**; (**e**) Preparation of (*E*)-β-farnesene analogues **48** through amide coupling between thiadiazole-5-carboxylic acids and substituted amines; (**f**) Metal-free synthesis of densely functionalized 1,2,3-thiadiazoles **49** via an intermolecular [3 + 2] heterocyclization; (**g**) Construction of 5-(4-cyclopropyl-5-((3-fluorobenzyl)thio)-4*H*-1,2,4-triazol-3-yl)-4-methyl-1,2,3-thiadiazole **50** from ethyl 4-methyl-1,2,3-thiadiazole-5-carboxylate.

Compounds such as 5-(4-methyl-1,2,3-thiadiazol-5-yl)-1,3,4-oxadiazole-2-thiol **43** and derivatives **44** (Scheme 8a) have been evaluated for their herbicidal activity, demonstrating significant effects against species such as *Brassica campestris* and *Echinochloa crusgalli* [59].

The combination of the heterocyclic rings of 1,2,3-thiadiazole and 1,3,4-oxadiazole in a single compound presents new opportunities for designing bioactive agents with potential agricultural applications. These results constitute an effective synthetic strategy that stands out not only for its efficiency but also for the diverse possible applications of the obtained compounds in the pharmaceutical and agricultural fields. The combination of conventional and microwave-assisted methodologies for synthesizing these compounds demonstrates a modern and practical approach that can be replicated in future research on bioactive compounds and their applications in crop protection and drug therapies. To identify new lead compounds with high biological activity, a series of 4-methyl-1,2,3-thiadiazole-5-carboxaldehyde benzoyl hydrazone **45** was designed and synthesized (Scheme 8b). Preliminary biological assay results showed that the compounds exhibit moderate to high fungicidal activity in vitro against six fungal species at 50 μg/mL. Furthermore, some of the compounds demonstrated significant curative activity against tobacco mosaic virus (TMV) in vivo at 500 μg/mL. A structure-activity relationship analysis for the compounds against *Valsa mali* revealed that those derivatives containing a halogen at the *para* position of the phenyl ring showed the best activity, with compound (R = 2,4-diCl), being particularly noteworthy, displaying a broad spectrum of fungicidal activity against various pathogens, including *V. mali*, *Botrytis cinerea*, *Pythium aphanidermatum*, *Rhizoctonia solani*, *Fusarium moniliforme,* and *Alternaria solani*, with EC_50_ values of 8.20, 24.42, 15.80, 40.53, 41.48, and 34.16 μg/mL, respectively. This study highlights the potential of 4-methyl-1,2,3-thiadiazole derivatives as antifungal and antiviral agents. It suggests that these compounds could help develop new treatments for fungal and viral diseases in plants, with applications in both medicine and agriculture [60]. *N*-(substituted-phenyl)-*N’*-(4-methyl-1,2,3-thiadiazole-5-yl)-thiourea-type compounds **46** have demonstrated applications in agriculture as pesticides and plant growth regulators. The synthesis was carried out via solid–liquid phase-transfer catalysis using PEG-600 (Scheme 8c), and the compounds were evaluated for their biological activities. Some showed moderate fungicidal activity and low growth-regulating activity in cucumber cotyledons. These results suggest that although the compounds have moderate potential as pesticides, further research is needed to optimize their biological efficacy, particularly for crop protection [61]. Another report presented the design and synthesis of a series of novel pyrazole oxime derivatives incorporating a 1,2,3-thiadiazole moiety. The synthesis was accomplished through a sequence of chemical transformations, including condensation reactions, reductions, and chlorinations. The resulting compounds exhibited remarkable insecticidal and acaricidal efficacy against *Plutella xylostella* and *Tetranychus cinnabarinus*, as well as notable antitumor activity [62]. These findings suggest that the new derivatives exhibit broad-spectrum bioactivity and hold considerable potential for applications in both agricultural and pharmaceutical fields.

To establish biochemical technology for inducing the production of valuable secondary metabolites, a new family of non-natural elicitors, **47**, derived from the plant activator benzo-1,2,3-thiadiazole-7-carboxylic acid, was designed and synthesized (Scheme 8d). These synthetic elicitors demonstrated potent induction activity in taxoid biosynthesis in *Taxus chinensis* cell cultures. Among the compounds evaluated, benzo-1,2,3-thiadiazole-7-carboxylic acid 2-(2-hydroxybenzoxyl)ethyl ester was significantly more effective than methyl jasmonate, previously considered the most potent chemical elicitor in this context, increasing the content and production of taxuyunnanine C by almost 40%. This family of novel elicitors induced defense responses in plants, including elevated H_2_O_2_ levels from the oxidative burst and activation of phenylalanine ammonia-lyase, which closely correlated with the remarkable stimulation observed in *T. chinensis* cell cultures. The obtained results suggested that the synthesized benzothiadiazoles may constitute a new class of elicitors capable of enhancing taxoid biosynthesis in plant cells, with potential implications for producing bioactive compounds of pharmaceutical interest [63]. In a related context, 15 (*E*)-β-farnesene analogues were obtained, among which compounds such as *N*-((*E*)-3,7-dimethyl-2,6-octadien-1-yl)-*N*-(2-naphthyl)-4-methyl-1,2,3-thiadiazole-5-carboxamide **48** (Scheme 8e) stand out, whose primary use is related to their activity as repellent and insecticidal agents, particularly against the aphid *Myzus persicae*. This offers an alternative to traditional insecticides in agriculture, a key consideration considering the growing concern over pesticide resistance. The main results showed that the synthesized compounds exhibited significant insecticidal activity against *M. persicae*, with mortality rates varying with compound structure, reaching up to 73.3% in some cases. These results suggested that (*E*)-β-farnesene analogs with 1,2,3-thiadiazoles have significant potential as pest control agents, contributing to more sustainable and environmentally friendly pest management strategies in agriculture [64]. These results may stem from the development of new compounds with high biological activity, which is crucial for pest control, particularly in the context of insecticide resistance. It is highlighted that the new analogs offer a balance of stability and efficacy, suggesting their potential as repellents and insecticides for controlling aphids, particularly *M. persicae*. The novel synthesized compounds exhibited superior repellent and insecticidal activity compared to the lead compound, (*E*)-β-farnesene. Some compounds achieved mortality rates of 80% or higher, indicating their potential utility in pest control. This advance is key in the search for more sustainable and effective alternatives to the challenges of pest management in agriculture [65].

A novel metal-free methodology has been developed for the formal intermolecular [3 + 2] heterocyclization between triethylammonium thioalkylamines and arylhydrazines, using a combination of I_2_ and O_2_ as efficient oxidants. This protocol provides concise, cost-effective access to novel, densely functionalized 1,2,3-thiadiazoles **49** (Scheme 8f), with yields ranging from good to excellent. The reaction is notable for its broad substrate spectrum, compatibility with diverse functional groups, mild conditions, and the use of environmentally friendly oxidants, facilitating flexible structural modifications [66]. Moreover, the synthesis of the compound 5-(4-cyclopropyl-5-((3-fluorobenzyl)thio)-4*H*-1,2,4-triazol-3-yl)-4-methyl-1,2,3-thiadiazole **50** was carried out starting from the key intermediate ethyl 4-methyl-1,2,3-thiadiazole-5-carboxylate (Scheme 8g). This intermediate reacted with hydrazine to give the corresponding carbohydrazide. Subsequently, the compound 5-(4-cyclopropyl-5-((3-fluorobenzyl)thio)-4*H*-1,2,4-triazol-3-yl)-4-methyl-1,2,3-thiadiazole was prepared by the reaction between a 1,2,4-triazole sulfide and 1-(chloromethyl)-2-fluorobenzene [67]. These compounds have high potential for biological activity studies due to their heterocyclic moieties, which have known pharmacological properties, such as 1,2,3-thiadiazoles and 1,2,4-triazoles, contributing to interactions with specific biological targets. The synthetic flexibility and functional compatibility offered by this methodology facilitate the design of derivatives with potential applications in the development of therapeutic or agrochemical agents.

## 4. Applications for Electronic Materials and Devices: Semiconductor Polymers, Photovoltaic Materials, and Optoelectronics

The study of functionalized compounds, such as 4,5-disubstituted 1,2,3-thiadiazoles, has gained relevance due to their diverse applications in medicine and agriculture, including their antibacterial, antiviral, and fungicidal properties. In this context, Mazur et al. reported a significant development by offering a reliable method for the identification and quantification of isomers by mass spectrometry, a crucial technique for the rapid differentiation of isomeric compounds [68]. 4,5-Functionalized derivatives of 1,2,3-thiadiazoles were studied by positive-mode electrospray ionization mass spectrometry (+ESI–MS/MS). The dominant fragmentation of the protonated derivatives involves the loss of N_2_, generating the [MH-N_2_]^+^ ion, which then loses SO_2_, sulfonylaryl radicals, or NH_3_, giving rise to characteristic fragments (*m*/*z* 183, 182, 166, among others). Intermediate thirene- or isothiazole-type structures were proposed for this ion, the latter being the most stable. The presence of these fragment ions is consistent across aromatic substituents, and it was confirmed that the fragmentation pathway does not change significantly with different amino groups at the 5-position. The specific fragments allow for easy identification of 1,2,3-thiadiazoles in (+)ESI–MS/MS spectra. In negative mode (−)ESI–MS/MS), the fragments are concentrated in the sulfonylaryl group, without providing clear information about the thiadiazole nucleus. Furthermore, under favorable conditions, 1,2,3-triazoles can be converted to 1,2,3-thiadiazoles, mimicking a condensed-phase reaction, although the reverse conversion could not be confirmed. The initial loss of N_2_ and the subsequent fragmentation pathways provide a clear template for identifying and studying 1,2,3-thiadiazoles by mass spectrometry [68]. Isomeric derivatives of 1,2,3-thiadiazoles with different amino groups at the 5-position (piperidinyl, pyrrolidinyl, morpholinyl, *N*,*N*-dimethylamino) and substituted sulfonyl groups (methyl, ethyl, 4-methylphenyl) were studied by (+)ESI-MS/MS mass spectrometry. The main fragmentation of the protonated 1,2,3-thiadiazoles involves the initial loss of N_2_, followed by cleavages at the sulfonylaryl group, generating characteristic ions. Substitution of the amino group at the 5-position does not significantly affect the fragmentation pattern, although derivatives with *N*,*N*-dimethylamine present simpler spectra due to lower fragmentation at the cyclic amine. Varying the sulfonyl substituent (alkyl or aryl) does not substantially change the fragmentation pathways; the loss of N_2_ and related fragments is maintained. Specific fragments (*m*/*z* 183, 182, 154, 150) confirm the structure of thiadiazoles and their stability. IRMPD spectroscopy combined with DFT calculations identified that the resulting ions after N_2_ loss adopt (more stable) isothiacazole rather than thirene structures, confirming previous theories. In negative (−)ESI-MS/MS mode, the charge is concentrated on the sulfonylaryl fragment, with no clear evidence of transformation of thiadiazoles to triazoles. However, the fragmentation showed common features suggesting a possible, but unconfirmed, partial isomerization. The loss of N_2_ and the subsequent fragmentation pathways are key to identifying and characterizing 1,2,3-thiadiazoles, while IRMPD spectroscopy confirms the nature of their main fragments [69].

**Figure 2:**
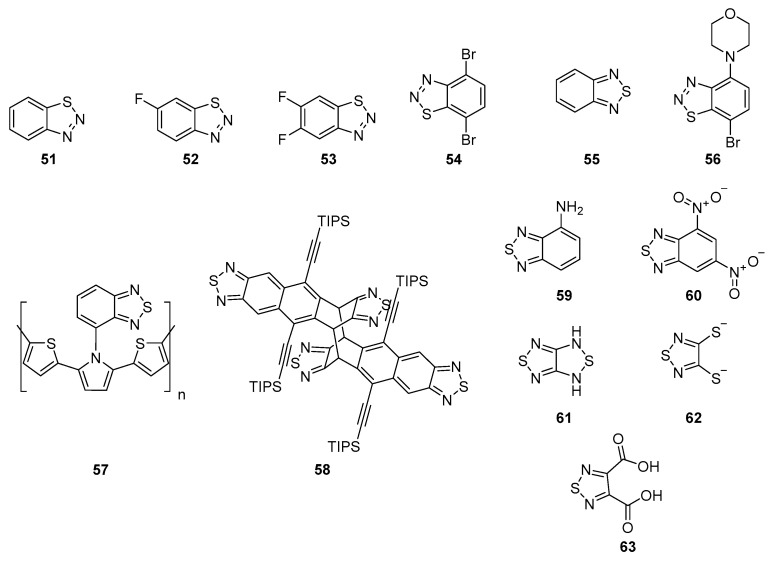
Structures of benzo[*d*]thiadiazole-containing compounds used in electronic materials.

Benzo[*d*][1,2,3]thiadiazole **51** (isoBT) and its halogenated derivatives, such as 6-fluoro-isoBT **52** and 5,6-difluoro-isoBT **53** (Figure 2), are described as a relevant material for the fabrication of semiconductor polymers for organic devices, including transistors, solar cells, photodetectors, and thermoelectric devices. IsoBT-derived polymers exhibit high charge mobilities and high-power conversion efficiencies in solar cells, positioning them as promising materials in the field of organic electronics [70]. The synthesis and structural characterization of **51** and its derivatives, as well as their use as building blocks for the fabrication of semiconducting polymers for organic electronic applications, were reported. The syntheses of **51** and its halogenated derivatives were followed by the characterization of the obtained compounds and polymers using techniques such as single-crystal and thin-film X-ray diffraction. Organic field-effect transistor (OTFT) devices and heterojunction solar cells (BHJ-OSC) were fabricated to evaluate the materials’ performance. The results showed that isoBT-based polymers achieved charge mobilities above 0.7 cm^2^ V^−1^ s^−1^ in OTFTs and conversion efficiencies of up to 9% in solar cells. Furthermore, fluorine functionalization was observed to enhance intermolecular interactions and structural organization, thus optimizing the device’s performance. The synthesis of 4,7-dibromobenzo[*d*][1,2,3]thiadiazole **54** from 2-aminobenzenethiol via a nitrosation reaction followed by an intramolecular cycloaddition was described. This compound is a key building block for photovoltaic materials and other organic electronic devices. This less-studied isomer of benzo[*c*][1,2,5]thiadiazole **55** was investigated to understand its electrochemical and optical properties. Cross-acyl reactions between **54** and thiophene derivatives were performed to optimize the reaction conditions and enhance the yields. The obtained products, *bis*(thienyl) compounds, were generated in moderate yields (40–61%). Regarding the optical properties, two absorption maxima were observed in the visible range, indicating intramolecular charge transfer in the compounds [18]. According to the promissory photovoltaic materials synthesis, these materials have applications in areas such as dye-sensitized solar cells, near-infrared organic materials, organic light-emitting diodes (OLEDs), and charge-transfer materials. The synthesis of 4-(7-bromobenzo[*d*][1,2,3]thiadiazol-4-yl)morpholine **56**, a novel compound that may be a valuable precursor for the development of photovoltaic materials, was achieved from 4,7-dibromobenzo[*d*][1,2,3]thiadiazole and morphine, and the synthetic protocol was carried out in different solvents and conditions, using triethylamine as the base to improve the product’s yield. The main results indicate that the desired compound was obtained in a maximum yield of 83% using DMSO as the solvent and triethylamine as the base. Furthermore, the reaction showed high selectivity, producing only the monosubstituted derivative [71].

Recent advances have demonstrated that 1,2,5-thiadiazole units serve as versatile, multifunctional structural elements that enhance the performance of materials in electronics, photonics, catalysis, and sensors, thereby consolidating their status as essential components in the next generation of innovative molecular materials. 1,2,5-Thiadiazoles constitute a family of aromatic heterocycles of great relevance in the design of functional materials due to their electron-withdrawing nature, their ability to modulate electronic and optical properties, and their remarkable chemical stability. Their versatility has allowed their incorporation into molecular and supramolecular architectures with applications in organic electronics, photonics, catalysis, and energy storage. A prominent example of the use of 1,2,5-thiadiazole units in electrochromic materials was reported by Wu and Li, where indene and benzothiadiazole units **57** were incorporated into the central pyrrole ring of a poly(thienylpyrrole) chain, yielding the polymers PDIT and PBDTA, along with their copolymers P(DIT-co-DTP) and P(BDTA-co-DTP). These electrochemically synthesized materials exhibited multicolor electrochromism (yellow, green, and blue) with high ΔT_max_ values (45.3% at 1314 nm and 43.5% at 930 nm) and superior coloration efficiencies (up to 504.6 cm^2^ C^−1^) in electrochromic devices (ECDs), while also displaying excellent electrochemical stability. These results demonstrate the potential of benzothiadiazoles as building blocks in π-conjugated polymers for advanced optoelectronic devices [72]. The introduction of terminal 1,2,5-thiadiazole groups has also been explored in dimerized acenic systems **58**. Using a one-pot protocol, layered electron acceptors (D_1_–D_4_) with 1,2,5-thiadiazole groups at the ends were obtained. Their crystal structures revealed a strong dependence of the photophysical and electrochemical properties on the conjugation length and molecular geometry. These compounds exhibited phenomena such as aggregation-induced emission (AIE) and intramolecular excimer emission (IEE), demonstrating the potential of thiadiazoles in the design of heteroacenes with emission and solid-state luminescent materials [73]. Charge-transfer (CT) complexes incorporating donor (D) and acceptor (A) units based exclusively on 1,2,5-thiadiazoles represent an innovative approach to generate materials with tunable electronic properties. CT complexes formed between 4-amino-2,1,3-benzothiadiazole **59** and acceptor derivatives such as 4,6-dinitro-2,1,3-benzothiadiazole **60** or [1,2,5]thiadiazolo [3,4-*c*][1,2,5]thiadiazole **61** were reported and characterized by XRD and UV-Vis spectroscopy. DFT and QTAIM studies revealed charge-transfer values up to 0.150 e and CT bands between 517 and 705 nm, indicating an intense donor–acceptor interaction mediated by hydrogen bonding and dispersion forces. These results consolidate the role of the 1,2,5-thiadiazole core as a dual electronic (D/A) module in the design of molecular materials with tunable optoelectronic properties [74].

The potential of 1,2,5-thiadiazoles extends to molecular catalysis. The complex [Bz-1-MeIm]_2_[Ni(tdas)_2_] (where tdas = 1,2,5-thiadiazole-3,4-dithiolate **62**), demonstrated outstanding activity in the electrocatalytic hydrogen evolution [75]. Electrochemical studies revealed two reversible redox couples (0.30 and −1.90 V vs. Ag/AgNO_3_) associated with the Ni^3+^/Ni^2+^ and Ni^2+^/Ni^+^ couples, and a marked catalytic activity in the presence of protons. In a neutral aqueous medium, the complex reached a TOF of 772.85 mol H_2_·mol^−1^·h^−1^ and a TOF of 1545.7, with a Faradaic efficiency of ≈101%, highlighting its superior performance compared to other Ni complexes under similar conditions. These properties are attributed to the high coordination and electron transfer capacity of the thiadiazolate ligand.

**Scheme 9:**
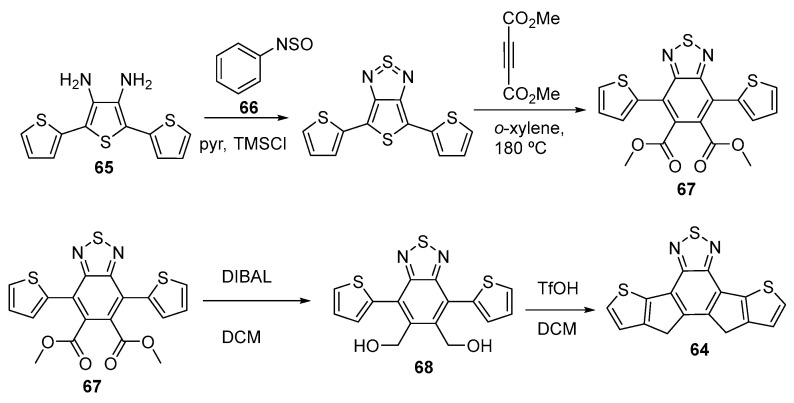
Synthesis of the methylene-bridged compound 10,11-dihydro-dithieno[3′,2′:2,3;2″,3″:6,7]-as-indaceno[4,5-*c*][1,2,5]thiadiazole **64**.

In the field of porous materials, the use of 1,2,5-thiadiazole-3,4-dicarboxylic acid **63** allowed the production of a red-luminescent europium microporous MOF, {[Eu_2_(H_2_O)L_3_]·3H_2_O}_n_, with excellent luminescent sensitivity towards nitrofuran antibiotics such as nitrofurantoin and nitrofurazone, showing high selectivities and response times less than 6 s. Furthermore, the microporous structure facilitated efficient CH_4_/C_2_H_2_ separation, demonstrating the synergy between the electronic character of the thiadiazole and the photophysics of the lanthanide, opening up new possibilities for chemical sensing and gas storage [76]. Finally, the design of π-conjugated donor–acceptor–donor (D–A–D) systems based on 1,2,5-thiadiazoles has allowed fine-tuning of the electronic properties. The methylene-bridged compound 10,11-dihydro-dithieno [3′,2′:2,3;2″,3″:6,7]-as-indaceno [4,5-*c*][1,2,5]thiadiazole **64** exhibits high planarity, a narrowed HOMO–LUMO gap, and an intense red emission at 638 nm (Scheme 9). The synthesis of compound **64** began with the reaction between [2,2′:5′,2′′-terthiophene]-3′,4′-diamine **65** and *N*-thionylaniline **66**, followed by treatment of the product formed with dimethyl but-2-ynedioate to form dimethyl 4,7-di(thiophen-2-yl)benzo[*c*][1,2,5]thiadiazole-5,6-dicarboxylate **67**. The reduction of this ester to the corresponding alcohol **68** was initially attempted using lithium aluminum hydride; however, the desired transformation did not occur, and no identifiable product could be isolated. Therefore, the reaction was carried out using diisobutylaluminum hydride (DIBAL) as a milder reducing agent, yielding the respective diol **68** in 82% yield. In the final step, it was reacted with trifluoromethanesulfonic acid (TfOH) in dichloromethane at room temperature to promote methylene bridge formation. Under these conditions, compound **64** was obtained in 53% yield after only 5 min of reaction. DFT calculations confirmed that the methylene bridge elevates the HOMO without affecting the LUMO, resulting in a bathochromic shift relative to its unbridged counterpart and a considerable Stokes shift, attributable to an intramolecular charge-transfer (ICT) state. This design strategy offers an effective method for tuning optical and electronic properties in next-generation functional organic materials [77].

## 5. Conclusions

Compounds based on thiadiazole and benzothiadiazole rings represent one of the most versatile and promising families of heterocycles in modern chemistry; this is due to the presence of sulfur and nitrogen atoms that confer exceptional chemical and biological properties. Among them, 1,3,4-thiadiazole systems have gained great relevance due to their outstanding antidiabetic activity, acting on key enzymes of carbohydrate metabolism and exhibiting inhibitory potency superior to that of conventional drugs such as acarbose. Furthermore, the variation in heteroatom positions within the ring in isomers such as benzo[*c*][1,2,5]- and benzo[*d*][1,2,3]-thiadiazoles determines their reactivity and stability, enabling their use in diverse fields such as bioimaging, biomarker detection, agriculture, and nanotechnology. Consequently, their structural capacity to generate fluorescent compounds, biosensors, and multifunctional materials consolidates them as key platforms for scientific and technological development.

Furthermore, advances in synthetic methods have enabled the development of more efficient, sustainable, and selective approaches to the synthesis of thiadiazoles and their derivatives. Procedures using *N*-tosylhydrazones, azides, or carbonyl compounds in the presence of elemental sulfur, combined with techniques such as photocatalysis or electrochemistry, have achieved higher yields and reduced the reactions’ environmental impact. These advances not only expand the available structural diversity but also facilitate the application of thiadiazoles in fields such as medicinal chemistry, pharmacology, and materials science. Overall, the combination of their remarkable biological potential, structural flexibility, and recent advances in synthesis positions thiadiazoles and benzothiadiazoles as essential building blocks for the design of new therapies, advanced electronic materials, and agricultural agents.

## Data Availability

Data presented in this study are included in the article. Further inquiries can be directed to the corresponding author.
